# Study of Heterogeneity of Ethylene/1-Octene Copolymers Synthesized with Zirconium and Titanium Complexes Bearing Diamine-bis(phenolate) Ligands

**DOI:** 10.3390/polym16030387

**Published:** 2024-01-30

**Authors:** Marzena Białek, Dominika Wiechoczek

**Affiliations:** Institute of Chemistry, University of Opole, Oleska 48, 45-052 Opole, Poland

**Keywords:** ethylene/1-octene copolymer, temperature rising elution fractionation, solvent/non-solvent fractionation, diamine-bis(phenolate) complex, FTIR, GPC, DSC

## Abstract

A series of ethylene/1-octene copolymers synthesized with diamine-bis(phenolate) complexes activated with Al(*^i^*Bu)_3_/[Ph_3_C][B(C_6_F_5_)_4_] were subjected to preparative temperature rising elution fractionation (TREF). The complexes used differed in the type of metallic center (Zr or Ti) or the amine donor in the pendant arm of the ligand (NMe_2_ or N*^i^*Pr_2_). The obtained fractions were then characterized via FTIR, DSC and GPC methods. It was found that all the copolymers had very broad chemical composition distributions, and the most heterogeneous was the copolymer produced by the titanium complex bearing a ligand with the N*^i^*Pr_2_ donor group. The difference in the comonomer incorporation into the macromolecules of the fractions was as high as 8.3 mol%. The melting temperature and molecular weight of the fractions changed nearly linearly with the increased elution temperature. Copolymers produced by zirconium catalysts were also fractionated by molecular weight using the solvent/non-solvent technique with subsequent analysis of the fractions. It was shown that the fractions have a similar composition, low molecular weight distribution and very broad comonomer distribution. Therefore, the comonomer content in the fractions was not a function of the molecular weight as was observed for the copolymers synthesized with the Ziegler–Natta catalysts.

## 1. Introduction

Polyolefins have been dominating the industrial production of polymers for many years due to their favorable properties, price and modification possibilities. A group of over 300 different commercial polyolefin products are available worldwide [1]. This group includes semi-crystalline copolymers of ethylene with higher 1-olefins, which are commercially sold as linear low-density polyethylene (LLDPE), very low-density polyethylene (VLDPE), olefin block copolymer (OBC) and (crystalline or amorphous) polyolefin elastomers (POEs), depending on their compositions and microstructures [2,3,4]. The incorporation of comonomer into polyethylene chains changes their structures and consequently reduces their crystallinity and extends the range of other polyethylene properties [3,4]. To define a copolymer, it is necessary to characterize both its molecular weight and molecular weight distribution (MWD) as well as the comonomer content and the chemical composition distribution (CCD) [5,6,7,8]. These parameters characterize the copolymers, and they are, to some extent, dependent on the copolymerization conditions. However, it is a catalyst used in the process that is crucial for the control of the polymer molecular weight and MWD, topology, stereochemistry, regiochemistry, type of end groups and, in the case of copolymerization, also for the control of the chemical composition and CCD [1,5,6,9,10,11]. Therefore, the relation between the catalyst and the product structure should be well understood for any group of catalysts used in the copolymerization process.

The molecular heterogeneity of ethylene/1-olefin copolymers produced by different types of metalloorganic catalysts is most commonly characterized by the use of fractionation techniques, which can be both analytical and preparative. The molecular weight heterogeneity can be analyzed with the use of gel permeation chromatography or solvent/non-solvent fractionation [12,13]. As regards compositional heterogeneity, temperature rising elution fractionation (TREF), crystallization analysis fractionation (CRYSTAF), crystallization elution fractionation (CEF) and high-temperature thermal gradient interaction chromatography (HT-TGIC) as well as thermal fractionation using successive self-nucleation and annealing (SSA) or step crystallization (SC) methods are employed [14,15,16,17,18,19,20]. However, preparative fractionation techniques, such as TREF and solvent/non-solvent fractionation, provide more information on the microstructure and length of copolymer chains than analytical ones since physically separated fractions are obtained and they can be further analyzed by other techniques [16]. Preparative fractionation enables the study of the relationship between the molecular weight and composition of the fractions, which, in addition to the CCD and MWD, is required for the full characterization of copolymers [16].

Matsko et al. [10] compared the profiles of the short-chain branching contents vs. molecular weights for ethylene/1-hexene copolymers synthesized over the supported Ziegler–Natta catalysts. The reported data showed a higher homogeneity of active sites in the vanadium–magnesium catalyst with respect to their copolymerization ability (despite a broader MWD) in comparison with the titanium counterpart. In turn, ethylene/1-octene copolymers, which were synthesized with the use of a constrained geometry catalyst, were found to have narrow composition distributions [9]. Interestingly, the molecular weights of the fractions derived from a copolymer with a lower comonomer content increased with the increasing elution temperature, while the molecular weights for the fractions coming from a copolymer with a higher comonomer content remained similar [9]. The studies of a series of 1-octene-based commercial Ziegler–Natta LLDPEs, which contained from 0.3 to 6.4 mol% of comonomer, revealed that the chemical compositions of the TREF fractions and their crystallinity are a function of the parent copolymer composition. Namely, the comonomer contents of similar TREF fractions increase with an increase in comonomer incorporation in the parent sample [21].

The family of group 4 metal complexes bearing amine bis(phenolate) ligands with a sidearm donor was introduced by M. Kol and co-workers and, originally, they were shown to be very active in the polymerizations of higher 1-olefins [22,23]. The application of such catalysts in ethylene/1-olefin copolymerization revealed their other features: good copolymerization ability and inability to uniformly insert the comonomer units into the polymer chains [24]. The high compositional heterogeneity of the produced copolymers was confirmed by their thermal fractionation with the use of the SSA method [24]. Thermal fractionation makes it possible to evaluate the compositional heterogeneity of polymeric chains [18]. However, as noted above, preparative fractionation followed by the characterization of each fraction by other methods gives more information on the molecular structures of copolymers. In the present work, ethylene/1-octene copolymers produced over diamine–bisphenolate complexes were fractionated according to their compositions using the preparative TREF method. The fractions were then subjected to FTIR, DSC and GPC analysis. For this study, we selected three copolymers with similar comonomer contents to evaluate the effect of the complex structure on the chemical composition distribution. Additionally, two copolymers with different compositions were used to assess the influence of the comonomer content on the CCD. In order to obtain more information on the relation between the molecular weights and compositions of copolymer chains, the selected copolymers were also fractionated on the molecular weight basis, using the solvent/non-solvent technique combined with GPC, DSC and FTIR.

## 2. Materials and Methods

### 2.1. Materials

2-(2-Butoxyethoxy)ethanol (Acros Organics, Geel, Belgium, 99+%), 2,6-di-tert-butyl-4-methylphenol (BHT, Acros Organics, 99%), o-dichlorobenzene (Acros Organics, 99%), C_6_D_4_Cl_2_ (99 atom % D, Deutero GmbH, Kastellaun, Germany), acetone (analytical grade, POCH, Gliwice, Poland) and methanol (Stanlab, Lublin, Poland, analytical grade) were used as received.

### 2.2. Characterization

The infrared spectra were recorded with the Nexus FTIR spectrometer (2002) produced by Thermo Nicolet Corporation (Madison, WI, USA). The polymer samples were prepared as KBr pellets or as a foil depending on the samples’ properties. Their scans were taken in the range from 4000 to 400 cm^−1^ with a 2 cm^−1^ resolution. The relative contents of unsaturated end groups were evaluated from the intensity ratios of the absorption bands with the maxima at 909 cm^−1^ (vinyl group), 965 cm^−1^ (*trans*-vinylene group) and 888 cm^−1^ (vinylidene group). To overcome the difference in the samples’ thickness, the intensities of bands were normalized using the absorption band at 2020 cm^−1^, which is proportional to the sample thickness. Comonomer content in parent copolymers and fractions was determined by FTIR spectroscopy. Estimation of comonomer content by FTIR spectroscopy was made in accordance with [25] using the appropriate calibration curve for ethylene/1-octene copolymers (Equation (1)), where *A_CH_*_3_ is the absorbance of the band at 1379 cm^−1^ after separation from the influence of the band at 1368 cm^−1^ and *A_CH_*_2_ is the absorbance of the band at 1368 cm^−1^ after separation from the influence of the band at 1379 cm^−1^.

(1) $$X\;(\%mol)=1.721\times {\left(\;\frac{A_{CH3}}{A_{CH2}}\right)}^{2}+4.0123\times \left(\;\frac{A_{CH3}}{A_{CH2}}\right)-0.0029$$

The melting temperatures (T_m_) and crystallinities (χ) of copolymers were determined via differential scanning calorimetry (DSC) with a 2010 DSC calorimeter from TA Instruments (New Castle, DE, USA) at the heating rate 10 °C/min in the range from 0 to 170 °C. The curves were registered after erasing the thermal history of polymers. The crystallinity (*Xc*) degree was calculated using the equation 2 [26]:

*Xc* = ΔH_f_ × (100/290) (2)

The molecular weight and molecular weight distribution (MWD) of polymers were measured by gel permeation chromatography using an Alliance 135 GPCV 2000 apparatus (Waters, Milford, MA, USA) equipped with HT3 (5 × 10^2^–3 × 10^4^ g/mol) and HT6E (5 × 10^3^–10^7^ g/mol) columns. Analysis was performed at 135 °C, using 1,2,4-trichlorobenzene (1.0 mL/min) as a solvent. The narrow molecular weight distribution polystyrene standards with a molecular weight range of 1000–3.7 × 10^6^ g/mol were used for calibration. 

### 2.3. Temperature Rising Elution Fractionation (TREF)

Preparative TREF was performed with the use of a PREP mc2 instrument produced by Polymer Char (Paterna, Spain). The weighted test sample of about 0.6–1.0 g and 100 mL of 1,2-dichlorobenzene stabilized with BHT were added to the vessel. After dissolution at 150 °C for 2 h, the temperature was decreased to 95 °C and the solution was held at this temperature for stabilization over 45 min. In the next step, i.e., crystallization, the temperature was decreased to 35 °C at a constant cooling rate of 0.1 °C. After stabilization at that temperature for 45 min, the first polymer fraction was collected. To assure that the whole amount of polymer corresponding to the fraction had been taken out from the vessel, two additional cycles with extra volumes of the solvent (2 × 50 mL) were performed. The precipitated polymer was dissolved again in a new portion of solvent at 50 °C. After stabilization for 45 min, the second fraction was collected. In order to obtain further fractions, the temperature was increased discontinuously up to 150 °C. In this way, the fractions at 60 °C (F3), 70 °C (F4), 80 °C (F5), 90 °C (F6), 100 °C (F7), 110 °C (F8) and 150 °C (F9) were collected in addition to the initial fractions obtained at 35 °C (F1) and at 50 °C (F2). The fractions were precipitated in an excess of acetone or methanol, filtered, washed with methanol and dried in a vacuum oven to a constant weight.

### 2.4. Solvent/Non-Solvent Fractionation

Fractionation by molecular weight was performed with the use of a PREP mc2 instrument produced by Polymer Char. The weighted test sample of about 1.0 g and 18 mL of 1,2-dichlorobenzene stabilized with BHT were added to the vessel. After dissolution at 150 °C for 60 min, the temperature was decreased to 120 °C and the solution was maintained at this temperature for stabilization over 30 min, after which 162 mL of non-solvent 2-(2-butoxyethoxy)ethanol stabilized with BHT was added. As recommended by the manufacturer, the temperature was increased to 124 °C and subsequently the instrument was cooled down to the fractionation temperature (120 °C) at the rate of 0.5 °C/min in order to achieve higher uniformity of the precipitated fraction. The solution was stabilized at this temperature for 40 min and then the first polymer fraction was collected. Successive fractions were obtained in the same way but the percentage of non-solvent (%vol) in the solvent/non-solvent mixture decreased. The shares of non-solvent in individual fractions were as follows: 90%vol (F1), 82%vol (F2), 74%vol (F3), 66%vol (F4), 58%vol (F5), 50%vol (F6) and 0%vol (F7). The fractions were precipitated in an excess of acetone, filtered, washed with acetone and dried in a vacuum oven to a constant weight.

## 3. Results and Discussion

### 3.1. Characterization of Parent Copolymers

**Figure 1:**
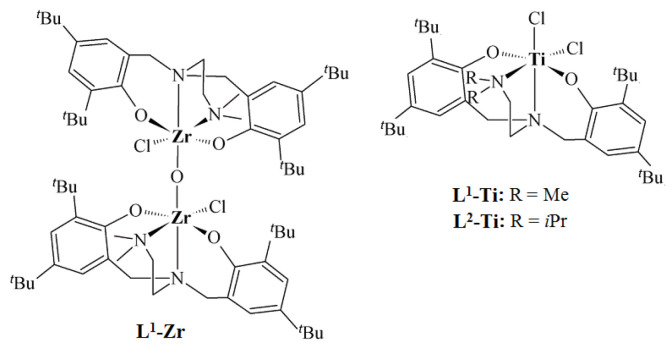
Structures of titanium and zirconium complexes with diamine-bis(phenolate) ligands [24].

Ethylene/1-octene copolymers used in the course of the studies were synthesized with diamine-bis(phenolate) complexes of zirconium and titanium (Figure 1), which were activated by Al(*^i^*Bu)_3_/[Ph_3_C][B(C_6_F_5_)_4_] [24]. The copolymers were synthesized at different comonomer concentrations. The data on the molecular weights, molecular weight distributions, compositions and end group characteristics of the investigated copolymers are summarized in Table 1**.**

The copolymers obtained with the zirconium catalyst had different compositions. The copolymers **C1** and **C2** contained 3.9% mol and 7.4% mol of 1-octene, respectively. Both those copolymers were characterized by low molecular weights and relatively narrow molecular weight distributions (**C1**:M_w_ = 14.6 × 10^3^ g/mol, M_w_/M_n_ = 3.2; **C2**:M_w_ = 28.0 × 10^3^ g/mol, M_w_/M_n_ = 3.1). The copolymer **C3** (3.1% mol) synthesized with the titanium complex bearing the same ligand as the zirconium catalyst had a similar chemical composition to that of **C1**. Furthermore, it was characterized by similar, i.e., relatively narrow, dispersity (M_w_/M_n_ = 3.3) and a higher molecular weight than **C1** (M_w_ = 222 × 10^3^ g/mol). The copolymer **C4**, which was synthesized with **L^2^-Ti**, contained 3.7% mol of 1-octene units. Its molecular weight was high (976 × 10^3^ g/mol) and its dispersity was relatively narrow (M_w_/M_n_ = 3.5). As can be seen from Table 1, the type and relative content of the unsaturated end groups in the copolymers are determined both by the comonomer incorporation level and by the catalyst that was used in the synthesis. The vinylidene end groups predominate in all copolymers, save for **C4** with the highest molecular weight of 976 × 10^3^ g/mol. The comonomer content in **C4**, however, was similar to that of **C1** and **C3**. This indicates that the presence of N*^i^*Pr_2_ instead of the NMe_2_ donor group in **L^2^-Ti** reduces the chance for the β-H elimination reaction to take place after the 1,2-insertion of the comonomer.

### 3.2. Fractionation of Copolymers Synthesized with L^1^-Zr

#### 3.2.1. Fractionation by Composition

**Figure 2:**
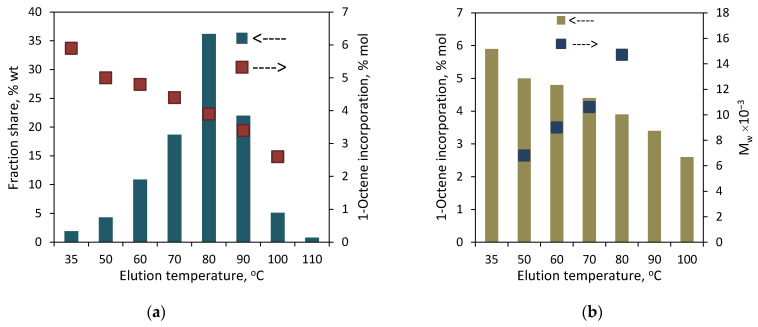
TREF results for the copolymer **C1**: (**a**) effect of elution temperature on shares of fractions and incorporation of 1-octene; (**b**) relation between elution temperature, comonomer incorporation and M_w_.

**Figure 3:**
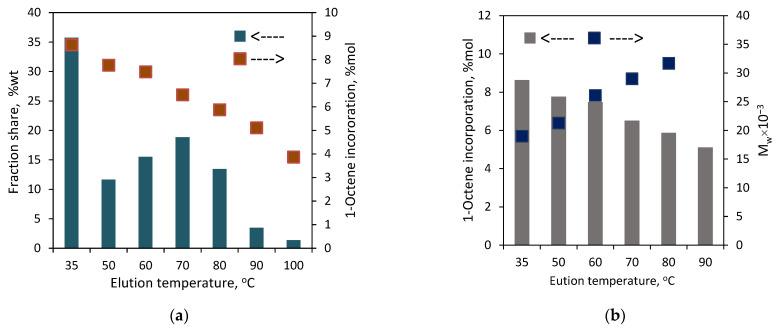
TREF results for the copolymer **C2**: (**a**) relation between elution temperature, shares of fractions and incorporation of 1-octene; (**b**) correlation between elution temperature, comonomer incorporation and M_w_.

The results of the preparative fractionation by composition for the copolymers synthesized with **L^1^-Zr** are presented in Figure 2 (for **C1**) and Figure 3 (for **C2**). They have also been attached in tabulated form in the Supplementary Materials (Table S1). As can be seen, both copolymers have heterogeneous compositions. In the case of **C1**, the fractions were collected at temperatures within the range from 35 to 110 °C (F1–F8). The fractions from F3 to F6 were dominating, and the largest share (36.2 wt%) was noted for F5, which was collected at 80 °C. In turn, the smallest share (0.8 wt%) was observed for F8. In the case of **C2**, which contained twice as much comonomer as **C1**, the fractions were obtained within 35 to 100 °C (F1–F7) and the first fraction was available at the highest share, i.e., ≈ 36 wt%. The comonomer content in the fractions of the copolymers decreased linearly with the increasing elution temperature. In the case of **C1**, the comonomer incorporation changed from 5.9 mol% for F1 to 2.6 mol% for F7 (very small amounts of fraction F8 made it impossible to determine the comonomer content). As regards the fractions of **C2**, the comonomer incorporation ranged from 8.6 mol% (F1) to 3.9 mol% (F7). It can be noticed that the comonomer contents in the fractions eluted at the same temperatures are different for both copolymers. The results are consistent with previous studies by Pasch and Ndiripo [21], which showed that the TREF fractions collected from different copolymers at the same elution temperature have different chemical compositions. Moreover, they found a direct correlation between the composition of the parent copolymer and the fractions: the comonomer content in similar TREF fractions increased with the increase in the comonomer content in the parent sample. In the case of the investigated copolymers, the different composition of fractions obtained at the same temperature may also be due to the much different molecular weights of the investigated copolymers. Although the comonomer contents in the fractions of **C1** obtained at a given temperature are lower than those in the fractions of **C2,** the molecular weight of **C1** is two times lower, which results in the better solubility of the fractions.

**Figure 4:**
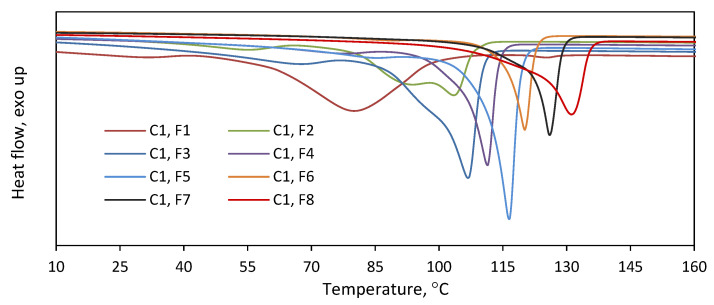
Thermograms recorded for fractions F1–F8 obtained in TREF fractionation of **C1**.

As is indicated by the above results, the copolymers were effectively separated into fractions with different comonomer contents. To check the homogeneity of the collected fractions, they were subjected to DSC analysis (Figure 4). The melting endotherms of all the fractions turned out narrower as compared to the endotherm of the parent copolymer (Figure S1). The fractions obtained at higher elution temperatures were homogeneous, with one narrow melting peak observed. In contrast, broader melting endotherms, also with multimodal patterns, were obtained for the fractions F1–F3. Moreover, the melting temperatures of the fractions were found to increase as a function of the elution temperature from F1 to F8. So, while the separation of macromolecules into fractions according to composition was not perfect, the subsequent fractions had significantly different compositions and thermal properties. The DSC analysis of the fractions of **C2** gave similar results (Figure S2).

**Figure 5:**
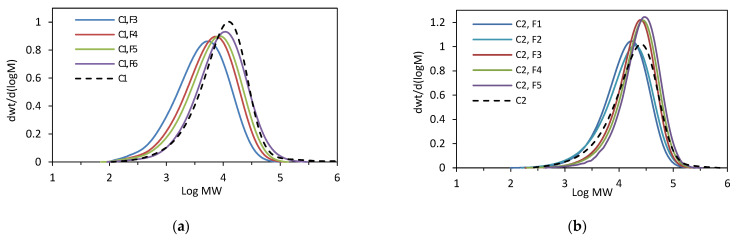
GPC curves: (**a**) for **C1** and its fractions (F3–F6); (**b**) for **C2** and its fractions (F1–F5).

To obtain more structural information about the tested copolymers, the relationship between the composition and molecular weight of the fractions was examined. The molecular weights of successive fractions of **C1** and **C2** increased linearly with the decreasing comonomer incorporation: from 6.8 × 10^3^ to 14.7 × 10^3^ g/mol for the F3–F6 fractions of **C1** (Figure 2b), and from 19.0 × 10^3^ to 31.7 × 10^3^ g/mol for the F1–F5 fractions of **C1** (Figure 3b). The molecular weight distribution values (M_w_/M_n_) for the fractions were similar or slightly lower as compared to the original sample (Figure 5).

#### 3.2.2. Fractionation by Molecular Weight

**Figure 6:**
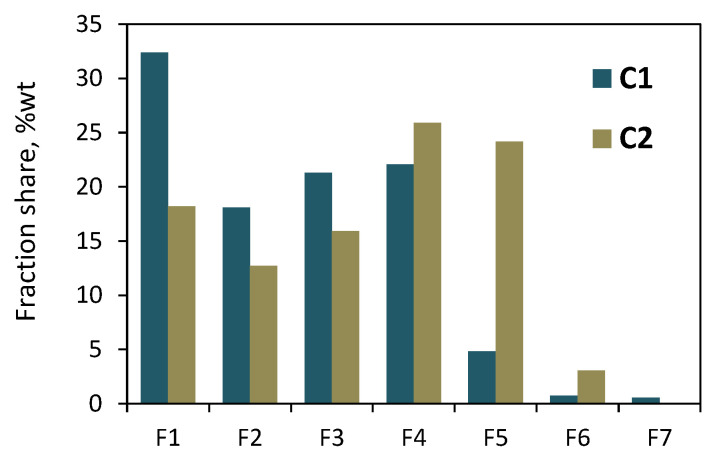
Results of fractionation by molecular weight of copolymers **C1** and **C2**: the effect of eluent composition on shares of fractions.

Both the copolymers that were synthesized with the zirconium complex were also subjected to solvent/non-solvent fractionation. The precipitation–dissolution procedure was repeated seven times, while the non-solvent content in the solvent/non-solvent mixture was varied between 90 and 0 vol%. Seven and six fractions were obtained for the **C1** and **C2** copolymers, respectively. The total mass of the collected fractions reached 93 wt% and 91 wt% of the initial mass of the sample of **C1** and **C2** subjected to fractionation. The results of the **C1** fractionations (Figure 6 and Table S2) showed the highest share of the first fraction (32.4 wt%). The amounts of subsequent fractions (F2–F4) were similar, from 18 to 22 wt%, while the fractions F5–F7 had very small shares—within 0.5–4.8 wt%. In the case of **C2**, the shares of the fractions F1–F5 ranged from 12.7 wt% to 25.9 wt%, while the share for the fraction F6 was the lowest—it was equal to 3.1 wt%.

**Figure 7:**
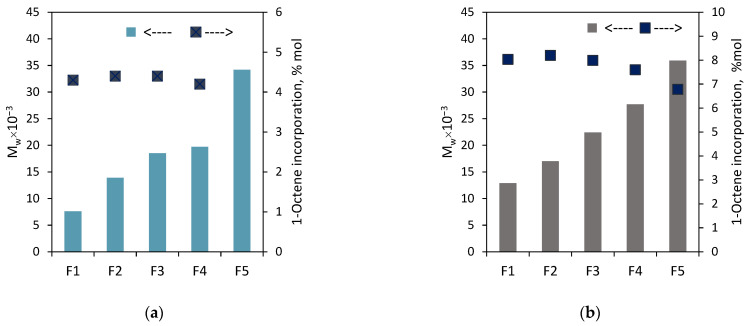
Effect of eluent composition on molecular weight and comonomer incorporation: (**a**) for the fractions of **C1**; (**b**) for the fractions of **C2**.

**Figure 8:**
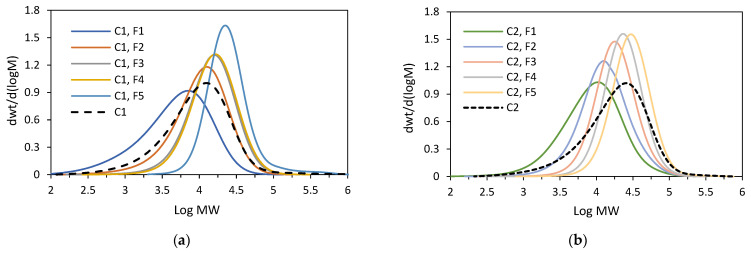
GPC curves: (**a**) for **C1** and its fractions (F3–F5); (**b**) for **C2** and its fractions (F1–F5).

As expected, the weight-average molecular weights of the fractions F1–F5 for both the copolymers increased approximately linearly (Figure 7) with the increasing solvent proportion in the solvent/non-solvent mixture: from 7.6 × 10^3^ g/mol to 34.2 × 10^3^ g/mol for the fractions of **C1** and from 12.9 × 10^3^ g/mol to 35.9 × 10^3^ g/mol for the fractions of **C2**. The molecular weights were not determined for F6 and F7 since their available amounts were very small. The dispersities of the fractions (Figure 8, Table S2) were lower as compared to the parent samples, and they were within the range 1.5–2.6 for the fractions of **C2**, while for the fractions F2–F5 of **C1**, they ranged from 1.7 to 2.2. The only exception was the fraction F1, for which the value was close to the dispersion of the parent sample.

**Figure 9:**
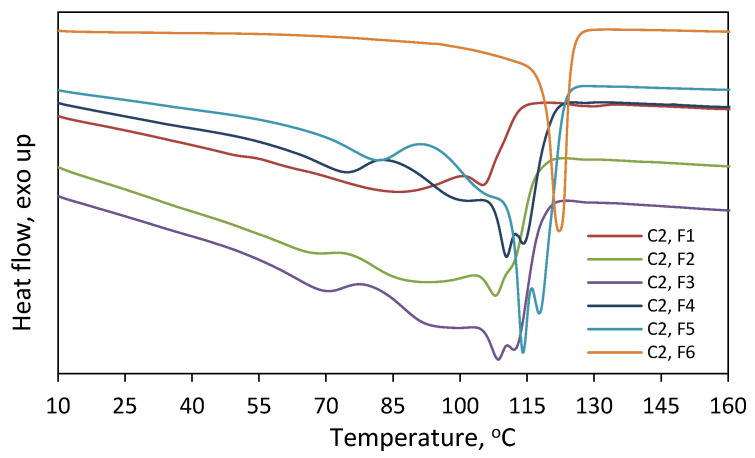
Thermograms recorded for fractions F1–F6 of **C2** obtained after fractionation by molecular weight.

On the other hand, it is seen in Figure 7 that the comonomer contents of the fractions F1–F4 are very similar. The incorporation of 1-octene into the F1–F4 fraction of **C1** and **C2** ranged from 4.2 to 4.4 mol% and from 7.6 to 8.2 mol%, respectively. In the case of the fraction F5 of **C2**, the incorporation was slightly lower, and it amounted to 6.8 mol%. The weight shares of the remaining fractions of the tested copolymers (Figure 6) were too small to determine their compositions. Those rather similar comonomer contents in the fractions (with a slight deviation for F5 of **C2**) confirm that fractionation primarily took place according to molecular weight and not according to chemical composition. Therefore, there is no relationship between the average molecular weight of the fraction and its average comonomer content, as was observed for the copolymers synthesized with the Ziegler–Natta catalysts, especially the titanium ones [10,27]. In addition, the thermograms obtained for F1–F5 of **C2** (Figure 9) were similar to those obtained for the parent copolymer **C2** (Figure S3) and they showed very broad, bi- and multimodal melting endotherms. According to earlier research [28], the appearance of several peaks in the differential scanning calorimetry indicates that the copolymer fractions are not uniform in composition. As can be seen, comonomer incorporation into shorter and longer chains (fractions F1–F5) was equally heterogeneous. The only exception is the fraction F6 obtained at the highest solvent-to-non-solvent ratio, which is quite homogeneous. Its high melting temperature (122.2 °C), which was only slightly lower than the T_m_ of PE obtained under the same conditions (≈128 °C [29]), proved that the fraction F6 contained macromolecules with low comonomer incorporation. However, its share in the copolymer was very small, around 3 wt%, which made it impossible to determine its molecular weight.

**Figure 10:**
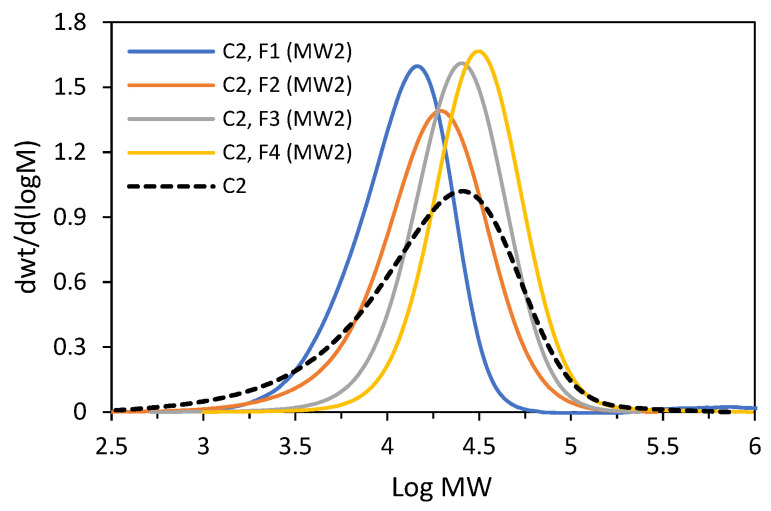
GPC curves recorded for **C2** and its fractions (F1–F4) obtained in second fractionation by molecular weight (MW2).

**C2** was subjected to fractionation by molecular weight again, with the proportion of solvent in the solvent/non-solvent mixture changed as compared to the first fractionation. The details are provided in the Supplementary Materials (Procedure S1). Five fractions were obtained from the experiment (Table 2). Four main fractions (F1–F4) were characterized by very narrow molecular weight distributions, ranging from 1.45 to 1.81 (GPC curves are shown in Figure 10). The fractions F1–F3 have very similar compositions with a comonomer incorporation equal to 7.3–8.0 mol%, while the incorporation for the fraction F4 was slightly lower, 6.4 mol%. However, all four factions were characterized by wide distributions of chemical compositions, which was confirmed by the DSC analysis (Figure S4). The DSC thermogram of the last fraction, i.e., F5, revealed a narrow melting peak at T_m_ = 122.6 °C, which is indicative of substantially homogeneous compositions of macromolecules. However, no further investigation of that fraction was possible since the available amount of the fraction was small (a few milligrams, 1.4 wt%). Those results confirm that the segregation process results mainly from the differences in the sizes of macromolecules, that the fractions have similar compositions and that they are very heterogeneous on account of a broad distribution of their chemical compositions. In addition to the main fractions, both the tested copolymers contained a more homogeneous fraction with a low comonomer content, but its share in the total mass of the polymer, i.e., from 0.8 to 3.0 wt%, was negligible.

The fractions of **C1** and **C2** were subjected to FTIR analysis to evaluate the relation between the molecular weight and the type and relative content of the unsaturated end groups, and thus the type of the chain termination reaction. The exemplary spectra are presented in Figure S5, and the results are summarized in Table 3. As can be seen from this table, the macromolecules of all the fractions of **C1**, i.e., of the copolymer with a lower comonomer content, are terminated either with the vinylidene or with the vinyl end groups. The vinylidene end groups are formed as the result of the 1,2-insertion of 1-octene followed by the chain termination reaction, which can occur due to β-hydrogen elimination and/or due to chain transfer to a coordinated monomer. In turn, the insertion of the ethylene molecule as the last one and subsequent mono- and/or bimolecular elimination of β-hydrogen results in vinyl unsaturation [30,31,32]. The mentioned termination mechanisms for the ethylene/1-octene copolymerization leading to unsaturated end groups are shown in Scheme S1 (reaction A–D). The content of the latter groups was considerably lower relative to the vinylidene end groups and their ratio was similar for all the fractions. As for the fractions of **C2**, i.e., of the copolymer with a higher comonomer content, the *trans*-vinylene end groups in addition to the vinylidene and vinyl end groups were present. They can be associated with the 2,1-insertion of a comonomer molecule followed by the mono- or/and bimolecular elimination of β-hydrogen (Scheme S1, reaction E and F) [30]. At the same time, the content of the vinyl end groups was relatively lower as compared to the fractions of **C1**. The ratio of the end groups (vinyl:vinylidene:*trans*-vinylene) in all the fractions, alike for the fractions of **C1**, was approximately the same. Therefore, there seems to be no relation between the type of chain termination reaction and the size of the macromolecules (i.e., molecular weight of fractions).

### 3.3. Fractionation by Composition of Copolymers Synthesized with Titanium Complexes

**Figure 11:**
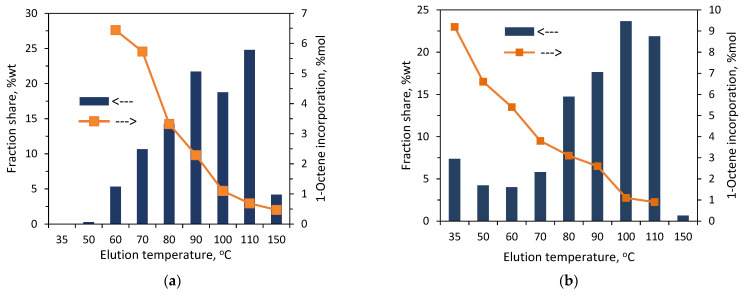
TREF results for the copolymers produced by titanium catalysts: (**a**) copolymer **C3**; (**b**) copolymer **C4**.

**Figure 12:**
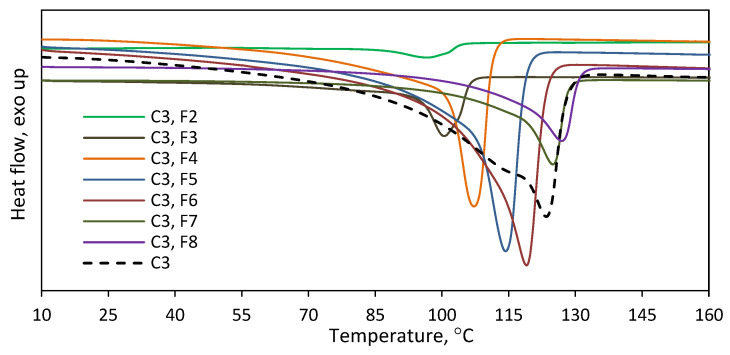
Thermograms recorded for fractions F2–F8 of **C3** obtained after TREF fractionation.

To find out the effect of the complex structure on the comonomer distribution, the copolymers **C3** and **C4** with similar comonomer contents (Table 1) were fractionated with the use of the TREF method. Those copolymers were prepared with the **L^1,2^-Ti** titanium complexes, which differed in the amine donor in the side arm of the ligand: NMe_2_ or N*^i^*Pr_2_. The results, as presented in Figure 11 and Table S3, showed the different chemical distribution for those copolymers. **C3** was split up into eight fractions. No fraction was obtained at the lowest elution temperature of 35 °C, and the share of the next fraction, which was collected at 50 °C, was negligible in practice, i.e., below 0.3 wt%. The shares of the other fractions ranged from 5.3 to 24.8 wt%. The difference in the comonomer contents between the fractions was almost 6 mol%, with the highest incorporation level of 6.4 mol% observed for F3 and the lowest level of 0.5 mol% noted for F9, which proves the significant heterogeneity of **C3**. The thermogram of the parent copolymer **C3** showed a wide temperature range for its melting peak, from ~30 °C up to ~135 °C. The melting ranges for the obtained fractions are narrower due to their lower heterogeneity in chemical composition (Figure 12). Moreover, the melting temperatures of successive fractions decrease linearly with the increasing comonomer content (Figure S6).

In the case of **C4**, the fractions were obtained at each elution temperature, i.e., it was separated into nine fractions. The highest shares, ranging from 14.7 to 23.7 wt%, were specific for the fractions F5 to F8. The shares for the fraction F1–F4 fell within the range of 4.0–7.4 wt%, and the lowest share, 0.7 wt%, was obtained for the fraction F9. As can be seen from Figure 11, there is a typical relation between the elution temperature and the 1-octene incorporation level as well as between the comonomer incorporation degree and the melting point of a fraction (Figure S6). For the increasing elution temperatures, the incorporation of the comonomer decreases, which leads to a higher melting point of the collected fraction. The difference in the comonomer incorporation into the macromolecules of the fractions was as high as 8.3 mol%. The highest and the lowest comonomer content, which amounted to 9.2 mol% and 0.9 mol%, were shown by the fractions F1 and F8, respectively. It was not possible to determine the composition of the macromolecules in the fraction F9 since the amount of that fraction was too small (3 mg). Summarizing, the simple variation in the side arm of the ligand plays an important role in the comonomer distribution. The **L^2^-Ti** complex, with the N*^i^*Pr_2_ donor group within its structure, produced significantly more heterogeneous copolymers than the **L^1^-Ti** complex, which contained the NMe_2_ amine donor.

## 4. Conclusions

It was shown that copolymers produced by diamine-bis(phenolate) complexes of group 4 transition metals (Zr or Ti) have very broad chemical composition distributions. Both the metal type and ligand structure had an influence on the CCD of copolymers, but the effect of the ligand was more significant. The copolymer produced by the titanium complex bearing a ligand with the N*^i^*Pr_2_ donor group exhibited a higher heterogeneity and different elution pattern than those produced with the complexes with the NMe_2_ group. The results of the FTIR, DSC and GPC analysis showed that the comonomer incorporation decreased with the increasing elution temperature and that the changes in the melting temperature and molecular weight of the fractions as a function of the elution temperature were linear. In turn, the fractions with very narrow molecular weight distributions, wide comonomer distributions and similar comonomer contents were obtained via solvent/non-solvent fractionation. Both strongly branched and weakly branched macromolecules existed within the whole range of molecular weights, which indicates that the copolymer chain termination reactions are equally likely to occur independently on comonomer incorporation. Therefore, the comonomer content in the fractions was not a function of the molecular weight as was observed for the copolymers synthesized with the Ziegler–Natta catalysts. More homogeneous fractions separated with the highest proportion of the solvent (≥90%) were the exception. However, their shares were negligible (they did not exceed 3 wt%).

## Figures and Tables

**Table 1:** Properties of ethylene/1-octene copolymers studied [24].

Sample	Complex ^a^	M_w_ × 10^−3^ [g/mol]	M_w_/M_n_	X ^b^ [%mol]	Absorbance Ratio		
					A_910_/A_2020_ (Vinyl Group)	A_888_/A_2020_ (Vinylidene Group)	A_965_/A_2020_ (*Trans*-Vinylene Groups)
**C1**	**L^1^-Zr**	14.6	3.2	3.9	0.089	0.328	0.0
**C2**	**L^1^-Zr**	28.0	3.1	7.4	0.168	0.714	0.162
**C3**	**L^1^-Ti**	222	3.3	3.1	0.166	0.160	0.151
**C4**	**L^2^-Ti**	976	3.5	3.7	0.592	0.349	0.205

^a^ Activator: Al(*^i^*Bu)_3_/[Ph_3_C][B(C_6_F_5_)_4_], molar ratio Zr (or Ti):Al:B = 1:15:1.5. ^b^ Comonomer content determined by FTIR.

**Table 2:** Results of second fractionation by molecular weight of **C2**.

Fractions	Solvent/Non-Solvent, mL/mL	Amount of Fraction, g	Share of Fraction, wt%	M_w_, g/mol	M_w_/M_n_	1-Octene Content, mol%
F1	36/144	0.322	35.0	13,800	1.50	7.3
F2	50/130	0.167	18.1	21,900	1.81	8.0
F3	65/115	0.250	27.1	28,500	1.46	7.5
F4	79/101	0.169	18.4	37,400	1.45	6.4
F5	94/86	0.013	1.4	nd ^1^	nd ^1^	nd ^1^
F6	108/72	0.0	-	-	-	-

^1^ Not determined due to very small amount of the fraction F6.

**Table 3:** Relative content of unsaturated end groups for **C1** and **C2** and their fractions obtained via fractionation by molecular weight.

Sample	Fraction	Absorbance Ratio			Ratio of the End Groups ^a^ (Vinyl: Vinylidene: *Trans*-Vinylene)
		A_910_/A_2020_ (Vinyl Group)	A_888_/A_2020_ (Vinylidene Group)	A_965_/A_2020_ (*Trans*-Vinylene Groups)	
**C1**	F1	0.175	0.475	0	0.37:1:0
	F2	0.125	0.379	0	0.33:1:0
	F3	0.120	0.328	0	0.37:1:0
	F4	0.083	0.280	0	0.30:1:0
**C2**	F1	0.342	1.472	0.248	0.23:1:0.17
	F2	0.270	1.027	0.223	0.26:1:0.22
	F3	0.230	0.873	0.183	0.26:1:0.21
	F4	0.205	0.703	0.181	0,29:1:0.25
	F5	0.130	0.474	0.097	0.27:1:0.20

^a^ The ratio of end groups in the copolymer calculated assuming that the number of vinylidene groups is equal to 1.

## Data Availability

Data are contained within the article and Supplementary Materials.

## Supplementary Materials

The following supporting information can be downloaded at: https://www.mdpi.com/article/10.3390/polym16030387/s1, Table S1: results of preparative fractionation by composition of **C1** and **C2** synthesized with **L^1^-Zr**, Figure S1: thermogram recorded for the ethylene/1-octene copolymer **C1**, Figure S2: thermograms recorded for fractions F1–F7 obtained in TREF fractionation of copolymer **C2**, Figure S3: thermogram recorded for the ethylene/1-octene copolymer **C2**, Table S2: results of preparative fractionation by molecular weight of **C1** and **C2** synthesized with **L^1^-Zr**, Procedure S1: experimental details of the second fractionation of **C2** according to molecular weight, Figure S4: thermograms recorded for fractions F1–F5 of **C2** obtained after fractionation by molecular weight (MW2), Figure S5: FTIR spectra of fractions obtained in the fractionation of copolymer **C1** by molecular weight: (a) full spectrum, (b) expanded region 1320–1400 cm^−1^ and (c) expanded region 800–980 cm^−1^, Table S3: results of preparative fractionation by composition of **C3** and **C4** synthesized with **L^1^-Ti** and **L^2^-Ti**, respectively, Figure S6: relationship between the melting temperature and the degree of comonomer incorporation for the fractions obtained in the TREF fractionation of the copolymer **C3** and the copolymer **C4**, Scheme S1: possible termination mechanisms for the ethylene/1-octene copolymerization leading to unsaturated end groups, Procedure S2: ethylene/1-octene copolymerization.
