# Supplementary material for: Study of Heterogeneity of Ethylene/1-Octene Copolymers Synthesized with Zirconium and Titanium Complexes Bearing Diamine-bis(phenolate) Ligands
Source: Polymers (Basel). 2024 Jan 30;16(3):387. doi: 10.3390/polym16030387 (PMC10857099; doi:10.3390/polym16030387)
Supplement: Supplementary file 1 [file polymers-16-00387-s001.zip › polymers-2827293-supplementary.pdf]

Supplementary Materials

Study of Heterogeneity of Ethylene/1-Octene Copolymers Synthesized with Zirconium and Titanium Complexes Bearing Diamine-bis(phenolate) Ligands

Marzena Białek * and Dominika Wiechoczek

Institute of Chemistry, University of Opole, Oleska 48, 45-052 Opole, Poland
* Correspondence: marzena.bialek@uni.opole.pl

Table of Contents

Results of preparative fractionation by composition of **C1** and **C2** synthesized with **L^1-Zr**	**Table S1**	p. 2
Thermogram recorded for the ethylene/1-octene copolymer **C1**	**Figure S1**	p. 2
Thermograms recorded for fractions F1-F7 obtained in TREF fractionation of copolymer **C2**	**Figure S2**	p. 2
Thermogram recorded for the ethylene/1-octene copolymer **C2**	**Figure S3**	p. 3
Results of preparative fractionation by molecular weight of **C1** and **C2** synthesized with **L^1-Zr**	**Table S2**	p. 3
Experimental details of the second fractionation of **C2** according to molecular weight	**Procedure S1**	p. 3
Thermograms recorded for fractions F1-F5 of **C2** obtained after fractionation by molecular weight (MW2).	**Figure S4**	p. 4
FTIR spectra of fractions obtained in the fractionation of copolymer **C1** by molecular weight: (a) full spectrum, (b) expanded region 1320-1400 cm^{-1} and (c) expanded region 800-980 cm^{-1}	**Figure S5**	p. 4
Results of preparative fractionation by composition of **C3** and **C4** synthesized with **L^1-Ti** and **L^2-Ti**, respectively	**Table S3**	p. 5
Relationship between the melting temperature and the degree of comonomer incorporation for the fractions obtained in TREF fractionation of the copolymer **C3** and the copolymer **C4**	**Figure S6**	p. 5
Possible termination mechanisms for the ethylene/1-octene copolymerization leading to unsaturated end groups	**Scheme S1**	p. 6
Ethylene/1-octene copolymerization	**Procedure S2**	p. 6

Table S1. **R**esults of preparative fractionation by composition of **C1** and **C2** synthesized with **L^1-Zr**

Fraction	Elution temp., °C	Amount of fraction, g	Fraction share, wt%	M_n,[a)] g/mol	M_w,[a)] g/mol	M_w/M_n [a)]	1-octene incorporation, mol% [b)]
**Fractions of the copolymer C1**							
F1	35	0.016	1.9	-	-	-	5.9 [c)]
F2	50	0.036	4.3	-	-	-	5.0
F3	60	0.091	10.9	2100	6800	3.2	4.8
F4	70	0.156	18.7	2900	9000	3.1	4.4
F5	80	0.302	36.2	3200	10600	3.3	3.9
F6	90	0.184	22.0	4600	14700	3.2	3.4
F7	100	0.043	5.1	-	-	-	2.6
F8	110	0.007	0.8	-	-	-	-
**Fractions of the copolymer C2**							
F1	35	0.258	35.7	7300	19000	2.6	8.6
F2	50	0.084	11.6	7300	21300	2.9	7.8
F3	60	0.112	15.5	12000	26100	2.2	7.5
F4	70	0.136	18.9	13600	29000	2.1	6.5
F5	80	0.097	13.4	16000	31700	2.0	5.9
F6	90	0.025	3.5	-	-	-	5.1
F7	100	0.010	1.4	-	-	-	3.9

[a)] determined by GPC, [b)] determined by FTIR, [c)] determined by ^{13}C NMR

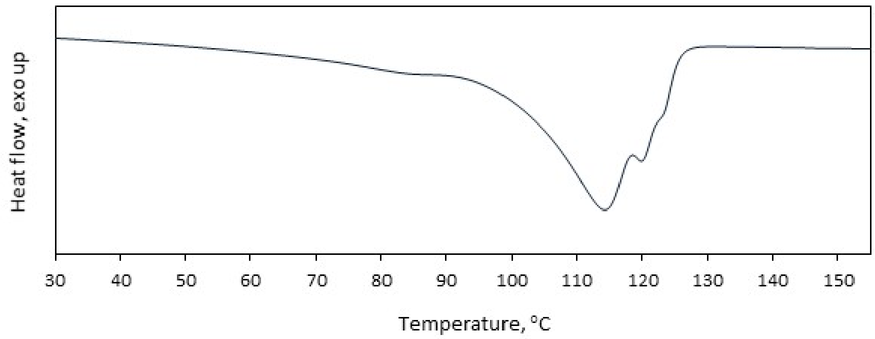

Figure S1. Thermogram recorded for the ethylene/1-octene copolymer **C1**.

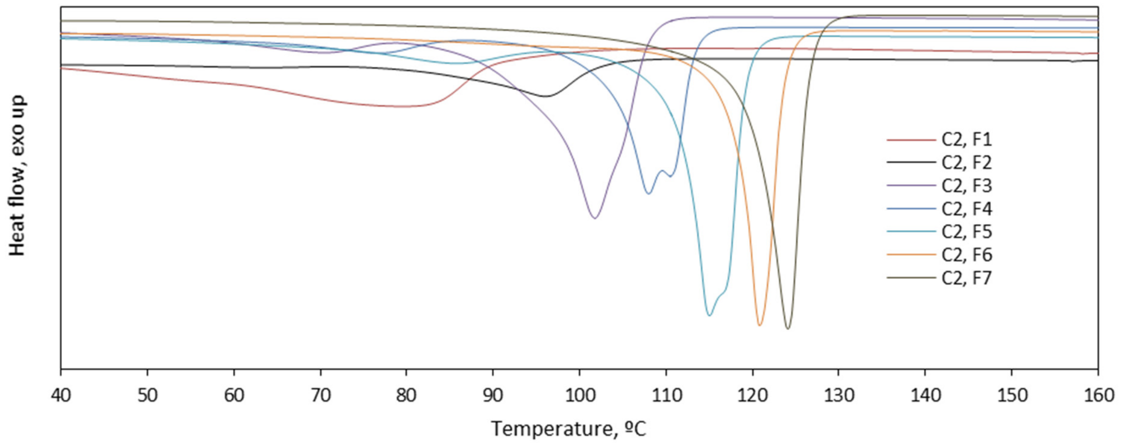

Figure S2. Thermograms recorded for fractions F1-F7 obtained in TREF fractionation of copolymer **C2**.

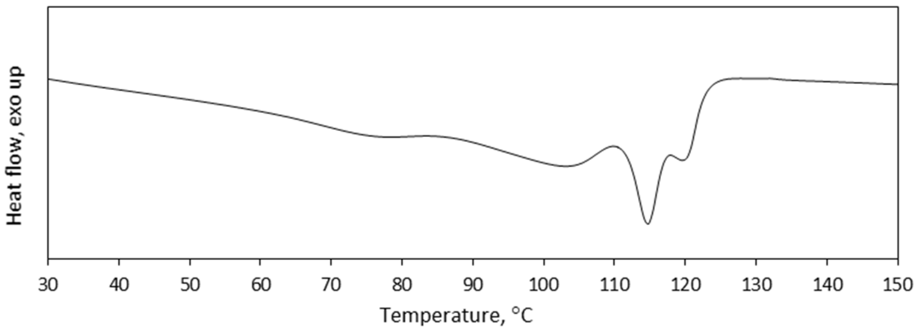

Figure S3. Thermogram recorded for the ethylene/1-octene copolymer **C2**.

Table S2. Results of preparative fractionation by molecular weight of **C1** and **C2** synthesized with **L^1-Zr**

Fraction	Solvent/non-solvent, ml/ml	Amount of fraction, g	Fraction share, wt%	M_n,[a)] g/mol	M_w,[a)] g/mol	M_w/M_n [a)]	1-octene incorporation, mol% [b)]
**Fractions of the copolymer C1**							
F1	18/162	0.301	32.4	2300	7600	3.3	4.3
F2	32/148	0.168	18.1	6300	13900	2.2	4.4
F3	47/133	0.198	21.3	10200	18500	1.8	4.4
F4	61/119	0.205	22.1	11400	19700	1.7	4.2
F5	76/104	0.045	4.8	19700	34200	1.7	-
F6	90/90	0.007	0.8	-	-	-	-
F7	180/0	0.005	0.5	-	-	-	-
**Fractions of the copolymer C2**							
F1	18/162	0.166	18.2	4900	12900	2.6	8.0
F2	32/148	0.116	12.7	8800	17000	1.9	8.1
F3	47/133	0,145	15.9	14000	22400	1.6	7.9
F4	61/119	0.236	25.9	18300	27700	1.5	7.6
F5	76/104	0.220	24.2	23900	35900	1.5	6.8
F6	90/90	0.028	3.1	-	-	-	-

[a)] determined by GPC, [b)] determined by FTIR

Procedure S1

Experimental details of the second fractionation of C2 according to molecular weight

Fractionation was performed with the use of a PREP mc2 instrument produced by PolymerChar. The weighted test sample of 1.0 g and 36 mL of 1,2-dichlorobenzene stabilized with BHT were added to the vessel. After dissolution at 150°C for 60 min, the temperature was decreased to 120°C and the solution was maintained at this temperature for stabilization over 30 min, after which 144 ml of non-solvent 2-(2-butoxyethoxy)ethanol stabilized with BHT were added. In order to achieve higher uniformity of the precipitated fraction, the temperature was increased to 124°C and subsequently the instrument was cooled down to the fractionation temperature (120°C) at the rate of 0.5°C/min. The solution was stabilized at this temperature for 40 min and then the first polymer fraction was collected. Successive fractions were obtained in the same way but the percentage of non-solvent (%vol) in the solvent/non-solvent mixture decreased. The shares of non-solvent in individual fractions were as follows: 80 %vol (F1), 72 %vol (F2), 64 %vol (F3), 56 %vol (F4), 48 %vol (F5) and 40 %vol (F6). The fractions were precipitated in an excess of acetone, filtered, washed with acetone and dried in a vacuum oven to a constant weight.

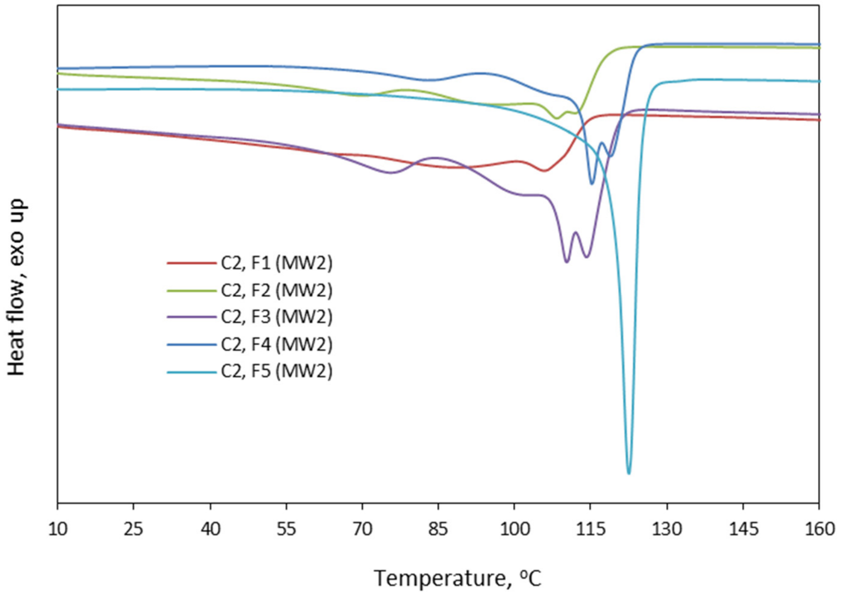

Figure S4. Thermograms recorded for fractions F1-F5 of **C2** obtained after fractionation by molecular weight (MW2).

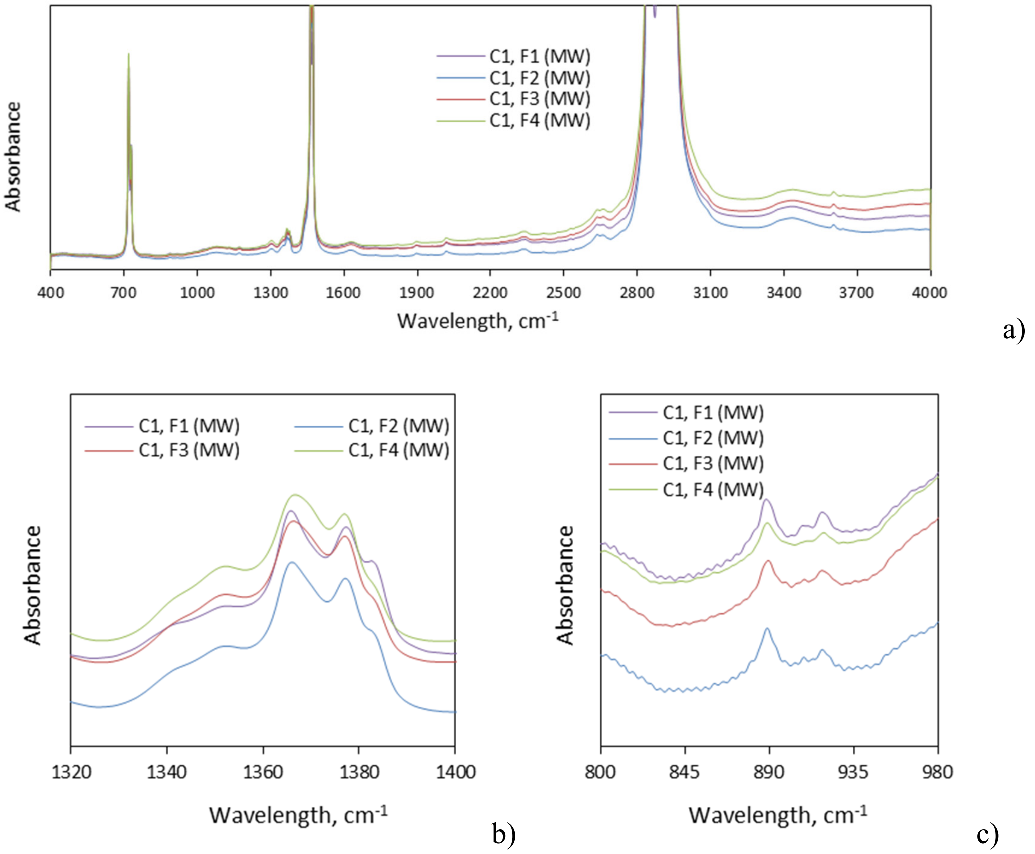

Figure S5. FTIR spectra of fractions obtained in the fractionation of copolymer **C1** by molecular weight: (a) full spectrum, (b) expanded region 1320-1400 cm^{-1} and (c) expanded region 800-980 cm^{-1} .

Table S3. Results of preparative fractionation by composition of **C3** and **C4** synthesized with **L^1-Ti** and **L^2-Ti**, respectively

Fraction	Elution temp., °C	Amount of fraction, mg	Fraction share, wt%	1-octene incorporation, mol% [a)]	T_m, °C [b)]	Crystallinity, % [b)]
**Fractions of the copolymer C3**						
F1	35	0	0	-	-	-
F2	50	0.002	0.3	nd [c)]	96.7	26.7
F3	60	0.038	5.3	6.4	100.5	32.0
F4	70	0.076	10.6	5.7	107.2	35.9
F5	80	0.102	14.3	3.3	114.3	39.0
F6	90	0.155	21.7	2.3	119.1	42.3
F7	100	0.134	18.8	1.1	125.0	42.3
F8	110	0.177	24.8	0.7	127.0	37.9
F9	150	0.030	4.2	0.5	nd [c)]	nd [c)]
**Fractions of the copolymer C4**						
F1	35	0.033	7.4	9.2	nd [c)]	nd [c)]
F2	50	0.019	4.2	6.6	87.8[d)]	-
F3	60	0.018	4.0	5.4	94.6	25.2
F4	70	0.026	5.8	3.8	100.4	26.7
F5	80	0.066	14.7	3.1	106.1	32.1
F6	90	0.079	17.6	2.6	113.5	31.5
F7	100	0.106	23.7	1.1	124.0	41.1
F8	110	0.098	21.9	0.9	126.5	36.8
F9	150	0.003	0.7	nd [c)]	nd [c)]	nd [c)]

[a)] determined by FTIR, [b)] determined by DSC, [c)] nd – not determined, [d)] small additional peak at 108.7°C

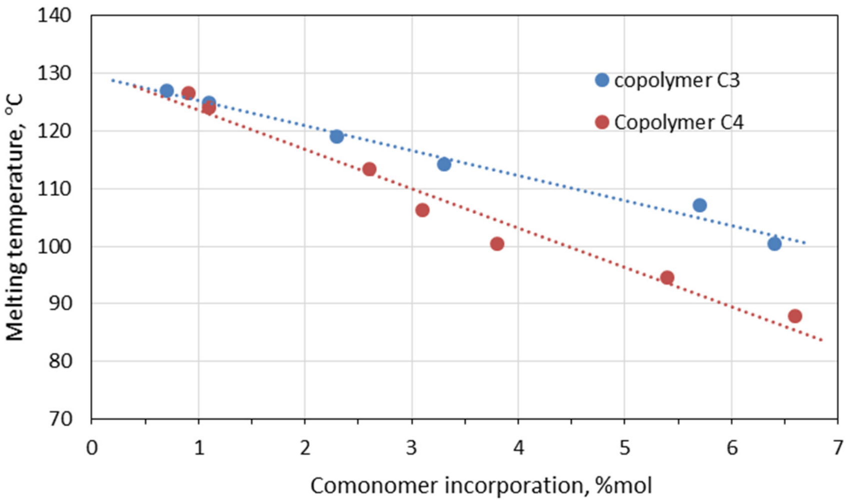

Figure S6. Relationship between the melting temperature and the degree of comonomer incorporation for the fractions obtained in TREF fractionation of the copolymer **C3** and the copolymer **C4**.

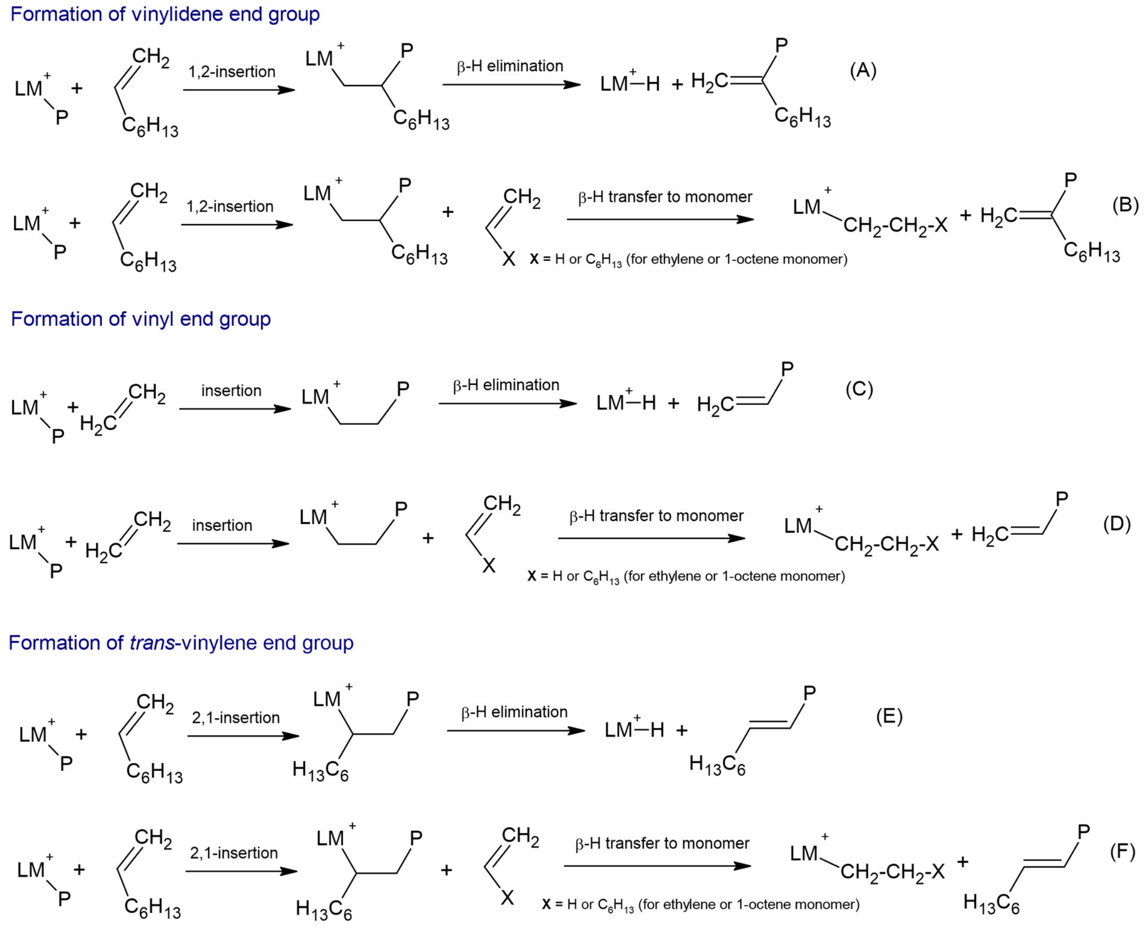

where: L - ligand, P - copolymer chain

Scheme S1. Possible termination mechanisms for the ethylene/1-octene copolymerization leading to unsaturated end-groups (*J. Polym. Sci.: Part A: Polym. Chem.* 2005, *43*, 2584–2597, Ref. [30]; *J. Polym. Sci. Part A: Polym. Chem.* 2000, *38*, 376-388, Ref. [31]; *J. Cat.* 2021, 400, 184–194, Ref.[32]).

Procedure S2

Ethylene/1-octene copolymerization

Copolymerizations were carried out at in Büchi glass reactor equipped with the magnetic stirrer and heating-cooling jacket using diamine-bis(phenolate) zirconium and titanium complexes activated with $Al(^{i}Bu)_3/[Ph_3C][B(C_6F_5)_4]$ as catalytic systems. They were conducted at 60°C for 30 min in hexane (150 ml) under a constant pressure of ethylene (5 bar) after the initial charge of liquid comonomer (from 1.8 to 20 ml). Molar ratio Zr (or Ti) : Al : B was equal to 1 : 15 : 1.5. Reactions were quenched with a dilute solution of hydrochloric acid in methanol. The copolymers were filtered, washed with methanol and dried in a vacuum oven to constant weight (*J. Polym. Sci., Part A: Polym. Chem.* 2017, *55*, 2467–2476, Ref. [24]).
